# Triple-negative ovarian apocrine carcinoma arising in a giant mature cystic teratoma: Case Report and Case Review

**DOI:** 10.3389/fmed.2026.1750157

**Published:** 2026-02-18

**Authors:** Inês Vaz, Teodor Svantesson, Manfred Kessler, Alexander Vogetseder, Andreas R. Günthert

**Affiliations:** 1 Gyn-Zentrum and Department of Gynecology and Obstetrics, Hirslanden Klinik St. Anna, Lucerne, Switzerland; 2 Department of Pathology, Cantonal Hospital of Lucerne, Lucerne, Switzerland; 3 Institute of Radiology and Nuclear Medicine, Hirslanden Klinik St. Anna, Lucerne, Switzerland

**Keywords:** apocrine carcinoma, case report, mature cystic teratoma, ovarian cancer, triple-negative

## Abstract

**Background:**

Mature cystic teratoma (MCT) of the ovary is one of the most common benign ovarian neoplasms in women of reproductive age. Malignant transformation is rare, occurring in approximately 1–2% of cases, and transformation into apocrine carcinoma is exceptionally uncommon. To date, only four such cases have been reported.

**Case presentation:**

We describe a 68-year-old woman with a giant ovarian tumor that had been slowly growing for over 40 years. Imaging revealed a 35 cm cystic mass, consistent with malignant degeneration and serum CA-125 was elevated (559 U/mL). The patient underwent exploratory laparotomy with complete removal of a 11 kg right ovarian tumor. Histopathological examination revealed a mature cystic teratoma of the ovary with malignant transformation into high-grade apocrine carcinoma. The tumor involved the cyst wall multifocally and showed no capsular rupture. Immunohistochemistry showed AR and EGFR positivity, ER and PR negativity, and HER2 score 2+ (FISH negative). The PET-CT showed 2 pericaval lymph nodes possible reactive after surgery. The postoperative course was uneventful, and from the outset the patient refused a comprehensive staging with hysterectomy, omentectomy and lymphadenectomy. At 6-month follow-up, CA-125 remained normal (<8 U/mL), with no evidence of recurrence.

**Conclusion:**

Ovarian apocrine carcinoma arising in MCT is exceedingly rare. Complete surgical excision remains the cornerstone of treatment, as no standard systemic therapy has been established. Further accumulation of similar cases is essential to better understand the biological behavior and optimize management of this rare tumor type.

## Introduction

Mature cystic teratoma (MCT), also known as dermoid cyst, represents 10–25% of all ovarian tumors and approximately 60% of benign ovarian neoplasms (1, 2). MCTs originate from a single germ cell and typically contain tissue from all three germ layers. Although mostly benign, malignant transformation occurs in only 1–2% of cases, most commonly into squamous cell carcinoma (2). Transformation into apocrine carcinoma is extremely rare, with only four cases reported to date (3–6).

Apocrine carcinoma usually arises in the breast or skin apocrine glands and accounts for less than 1% of cutaneous malignancies and approximately 1% of breast cancers (7–10). Histologically, apocrine carcinoma is characterized by large eosinophilic cells with nuclear pleomorphism and decapitation secretion. Immunohistochemically, apocrine carcinoma typically expresses androgen receptor (AR) and epidermal growth factor receptor (EGFR) and lacks estrogen (ER) and progesterone (PR) receptor expression (9, 10). We report a rare case of a triple-negative ovarian apocrine carcinoma arising in a giant mature cystic teratoma, with an unusually long clinical history.

## Patient information

A 68-year-old postmenopausal woman presented with progressive abdominal distension from a slowly enlarging mass present for over 40 years, which had grown more rapidly over the past eight years. Imaging in 2017 revealed a 14 cm ovarian mass suspicious for a teratoma, but the patient was reluctant to surgery and was lost to follow-up. She denied pain, weight loss, or changes in bowel or urinary habits, and had no relevant personal or family history of gynecologic malignancy (see Table 1).

**Table 1:** Case timeline.

Timepoint	Event
Initial presentation	Patient noticed abdominal distension and discomfort; imaging revealed a very large and suspicious right adnexal mass
Preoperative evaluation	CT scan showed a 35 × 25 × 28 cm heterogeneous mass with solid components; CA-125 mildly elevated
Surgery	Laparotomy and bilateral adnexectomy; patient declined hysterectomy, omentectomy, and lymphadenectomy
Postoperative course	Uneventful recovery Histopathology confirmed apocrine adenocarcinoma arising in mature cystic teratoma; triple-negative (ER−/PR−/HER2−), AR+, EGFR+
Follow-up	Patient under close surveillance; no adjuvant therapy administered due to early stage and patient preference

## Clinical findings and diagnostic assessment

On physical examination, abdominal distension was evident, with a large, firm, non-tender pelvic-abdominal mass extending to the xiphoid process. Laboratory investigations revealed an elevated serum CA-125 of 559 U/mL. Other tumor markers (CEA, CA19-9, AFP, SCC) were within normal limits. Breast examination and mammography were unsuspicious.

**Figure 1:**
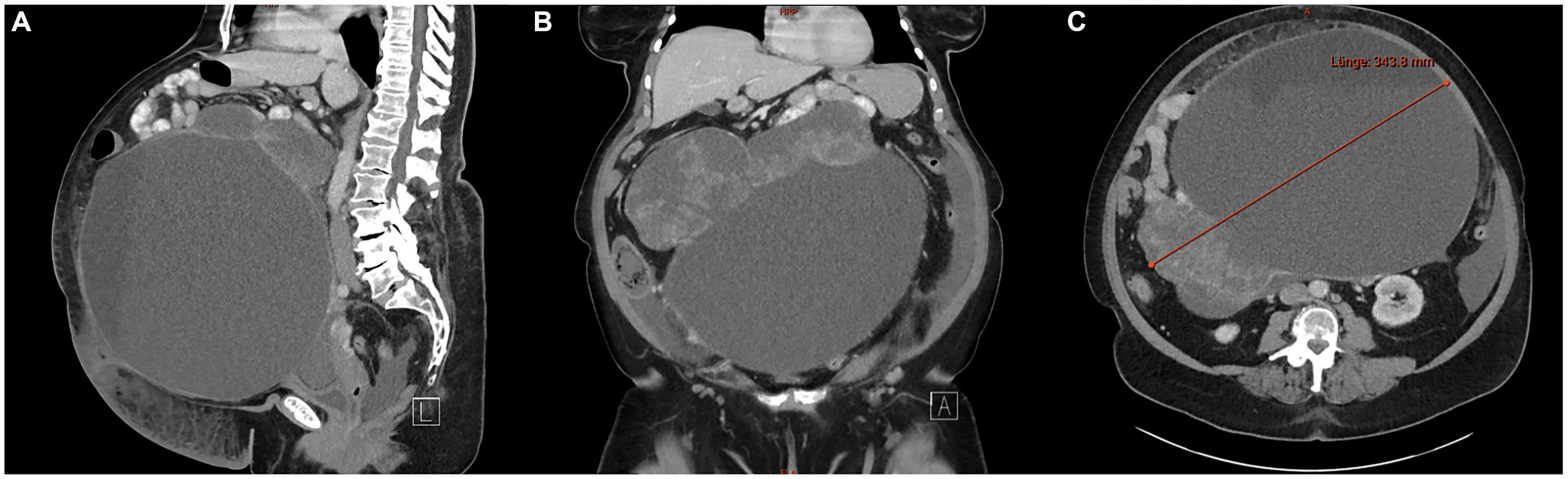
Contrast-enhanced computed tomography (CT) of the abdomen and pelvis reveals a giant, predominantly cystic right ovarian mass measuring approximately 35 cm in diameter, with multiple septations and fat-fluid levels, suggestive of a teratomatous lesion. **(A)** Sagittal view; **(B)** coronal view; **(C)** axial view.

Contrast-enhanced computed tomography (CT) of the abdomen and pelvis revealed a giant, predominantly cystic right ovarian mass measuring approximately 35 cm in diameter, containing multiple septations and fat-fluid levels, consistent with malignant degeneration/transformation of a known right ovarian teratoma. Small amount of ascites present. Pathologically enlarged lymph nodes retrocaval on the right and prominent cardiophrenic lymph nodes bilaterally were observed (Figure 1)

## Therapeutic intervention

**Figure 2:**
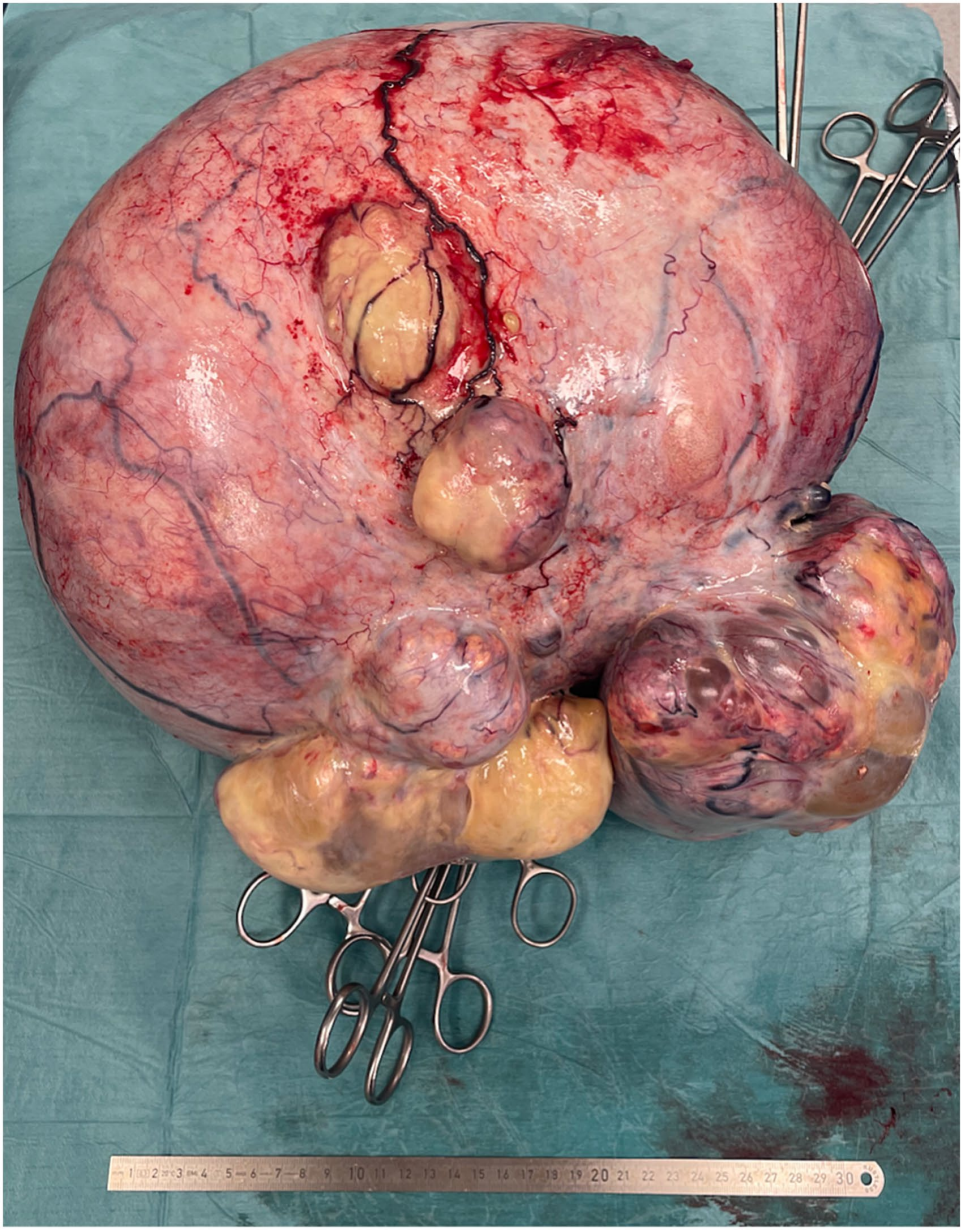
Surgical specimen showing the giant ovarian cystic teratoma, measuring 35 × 35 × 25 cm and weighing 11 kg. The external surface is smooth and intact.

The patient underwent exploratory laparotomy, which revealed a large right ovarian cystic tumor measuring 35 × 35 × 25 cm and weighing 11 kg (Figure 2). The tumor was completely excised without rupture. No enlarged lymph nodes, omental deposits, or peritoneal implants were identified intraoperatively. Peritoneal cytology was negative for malignant cells. The patient declined intraoperative frozen section and comprehensive surgical staging, including hysterectomy, contralateral oophorectomy, and lymph node dissection.

## Histopathological findings

Histopathological examination revealed a mature cystic teratoma of the right ovary with malignant transformation into high-grade apocrine carcinoma. The tumor involved the cyst wall multifocally, measured 35 cm in maximum diameter, and showed no capsular rupture. Grossly, the tumor consisted of cystic areas filled with sebaceous material and hair. Microscopically, the cyst wall contained mature ectodermal elements, including skin and adnexal structures, consistent with a mature cystic teratoma. Multiple solid nodules within the cyst wall demonstrated adenocarcinomatous transformation with extensive necrosis. The tumor cells displayed abundant eosinophilic cytoplasm, pleomorphic nuclei, and prominent nucleoli.

**Figure 3:**
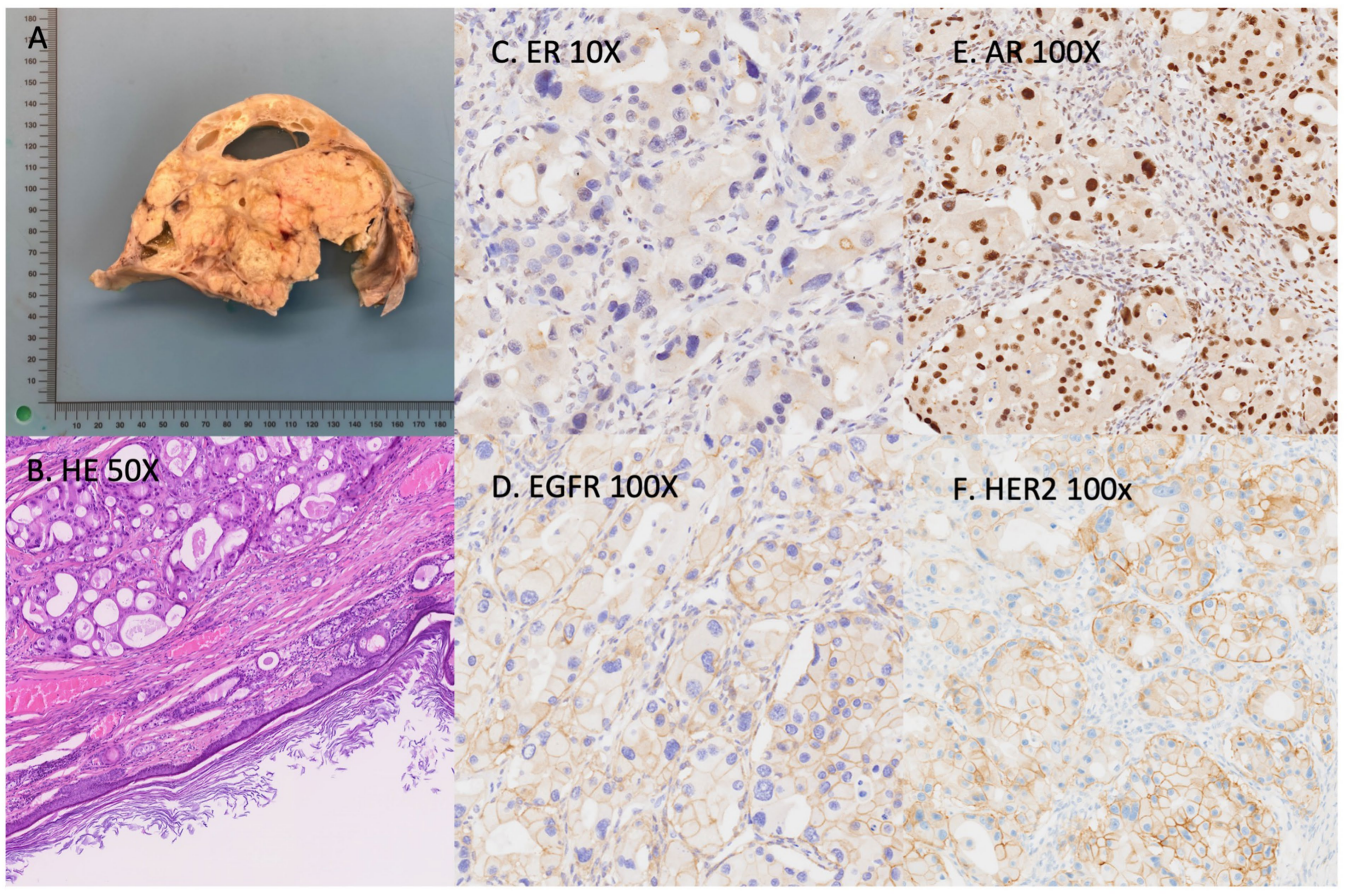
**(A)** Cut surface of the ovarian cyst showing extensive necrosis and cystic change. **(B)** Hematoxylin & eosin (H&E, 50×) demonstrating adenocarcinoma composed of large atypical cells with abundant eosinophilic cytoplasm and prominent nucleoli arising within the cyst wall. **(C)** Immunohistochemistry: ER-negative staining. **(D)** Immunohistochemistry: tumor cells positive for EGFR (100×). **(E)** Tumor cells positive for androgen receptor (AR 100×). **(F)** HER2 immunostaining shows a score of 2+. (AR, androgen receptor; ER, estrogen receptor; EGFR, epidermal growth factor receptor).

Immunohistochemical staining revealed positivity for androgen receptor (AR), cytokeratin 7 (CK7), and epidermal growth factor receptor (EGFR); partial weak positivity for GATA3; and negativity for estrogen receptor (ER), progesterone receptor (PR), prostate-specific antigen (PSA), paired box gene 8 (PAX8), cytokeratin 20 (CK20), SOX10, and thyroid transcription factor-1 (TTF1). HER2 immunostaining was 2+, fluorescence *in situ* hybridization (FISH) showed no gene amplification. The Ki-67 proliferation index reached 50% (see Figure 3).

These findings supported the diagnosis of adenocarcinoma with apocrine differentiation arising within a mature cystic teratoma. Histopathological examination of the contralateral ovary and fallopian tube showed no evidence of malignancy.

## Postoperative course and follow-up

The postoperative recovery was uneventful, and the patient was discharged on postoperative day 3. Five weeks postoperatively, positron emission tomography (PET) revealed two mildly enlarged para-aortic lymph nodes (9–10 mm), considered indeterminate for malignancy.

At one month follow-up, CA-125 had normalized to 7.9 U/mL. The patient remains under close surveillance without evidence of recurrence at six months postoperatively, and serum tumor markers remain within normal limits. She declined comprehensive staging surgery and the option of adjuvant treatment (see Table 1).

## Discussion

Mature cystic teratomas (MCTs) are the most common ovarian germ cell tumors, accounting for approximately 10–20% of all ovarian neoplasms (1, 8, 10). Although typically benign, malignant transformation (MT) occurs in only 1–3% of cases (3). Because all three germ layers are present, various malignancies may develop, including adenocarcinomas, malignant struma ovarii, carcinoid tumors, melanomas, and sarcomas. The most frequent malignant evolution, however, is squamous cell carcinoma (SCC) derived from the ectoderm, which represents the majority of MT-MCTs (1).

The exact mechanism of malignant transformation in MCT is unclear. Considering that 80% of MCTs are diagnosed during reproductive age, malignant transformation appears related to the long-term persistence of an unremoved teratoma into postmenopausal years (1, 8, 10). Chronic inflammation, hormonal influences, or accumulation of genetic mutations over time have been proposed as potential contributors.

Preoperative diagnosis of a benign MCT is relatively straightforward, as imaging modalities can detect characteristic features such as fat, calcification, bone, and teeth (1, 10). In contrast, preoperative diagnosis of malignant transformation remains difficult. Certain gross changes may raise suspicion—such as wall thickening, mural nodules, necrosis, hemorrhage, or adherence to surrounding structures—but definitive diagnosis is typically achieved only after histopathological examination (1).

The clinical usefulness of tumor markers in detecting malignant transformation is limited. CA-125 may be elevated, as in the present case, but this finding is nonspecific. Since germ cell and epithelial tumors cannot always be distinguished preoperatively, it is advisable to include CA-125 and other epithelial markers in the preoperative evaluation (9, 11).

## Apocrine adenocarcinoma arising in MCT

Transformation into apocrine adenocarcinoma is exceptionally rare (3–6). To date, only a handful of such cases have been documented worldwide the first authentic case of apocrine adenocarcinoma of the ovary was described by Morimitsu et al. (6). More recently, Holmes and Robb (4) reported another case of ovarian apocrine adenocarcinoma with supraclavicular lymph node metastasis, although details on pelvic or abdominal spread, treatment, and prognosis were limited. Xu et al. (5) reported a third case, the first known case of an apocrine adenocarcinoma arising within an ovarian mature cystic teratoma with pelvic lymph node metastasis. Recently, Hirofumi (3) reported a fourth case involving synchronous primary ovarian and breast cancers. It was difficult to determine whether the ovarian cancer was primary or metastatic until the surgery was performed (Table 2).

**Table 2:** Previously reported ovarian apocrine adenocarcinoma cases.

Case	Year, author	Age (years)	Stage/Spread	Treatment	Outcome	Reference
1	1993, Morimitsu	41	5 cm	Surgery	Limited follow-up	(6)
2	2018, Holmes	32	4 cm initially FIGO Ia	Surgery	Supraclavicular lymph node 6 months after surgery	(4)
3	2018, Xu	46	12 cm, pelvic lymph node, solid component adhered to rectum, FIGO IIIc	Surgery, chemo sixth cycle of TP regimen	Advanced disease, chemoresistant	(5)
4	2025, Hirofumi	44	Left ovary MCT 14 cm; no nodal metastasis Synchronous breast cancer	Hysterectomy + BSO + omentectomy + LN dissection	Post-op favorable, no adjuvant chemo	(3)
5	Present case	57	Right ovarian MCT; patient declined full staging	Surgery (ovarian excision)	Uneventful post-op; under surveillance	This report

MCT, mature cystic teratoma; BSO, bilateral salpingo-oophorectomy; LN, lymph node.

Due to the paucity of cases, the biologic behavior and metastatic potential of ovarian apocrine adenocarcinoma remain uncertain. Reported cases of malignant transformation in MCT suggest disease typically remains confined to the pelvis, with distant metastases being rare. Whether apocrine differentiation confers a greater risk of lymphatic or distant dissemination is still unclear (2).

## Comparison with breast and cutaneous apocrine carcinoma

Literature on apocrine adenocarcinoma of the breast is more extensive. Treatment strategies are like those used for invasive ductal carcinoma, including surgery, chemotherapy, radiotherapy, and endocrine therapy, with comparable prognosis (7, 9, 12, 13). Cutaneous sweat gland adenocarcinomas, particularly those of apocrine or eccrine type, are typically locally invasive and may metastasize to regional lymph nodes. The likelihood of metastasis and recurrence correlates with the malignancy of the primary lesion. Nevertheless, whether these features apply to apocrine adenocarcinoma arising within an ovarian teratoma is uncertain due to limited literature (12).

## Immunophenotypic and molecular characteristics

Apocrine carcinoma usually demonstrates a triple-negative immunophenotype (ER−, PR−, HER2−) with AR and EGFR positivity, as seen in this case. AR expression implies potential sensitivity to antiandrogen therapy, though resistance mechanisms—such as ARv7 splice variants and AR/NCOA2 co-amplification—may limit clinical efficacy (3, 7, 9, 14).

In breast cancer, AR-positive apocrine carcinoma has shown favorable prognosis even without adjuvant chemotherapy, supporting possible treatment de-escalation strategies in early-stage disease (14).

According to the 2019 WHO Classification of Breast Tumors, apocrine carcinoma (termed *carcinoma with apocrine differentiation*) is defined morphologically by >90% of tumor cells displaying apocrine features and an ER−/AR+ receptor profile (15). When strictly defined, apocrine carcinoma represents ~1% of all breast cancers, making its occurrence in the ovary extraordinarily rare.

## Management and prognosis

The mainstay of treatment for MCT with malignant transformation is surgical resection. For early-stage (FIGO IA) disease, conservative unilateral oophorectomy without adjuvant therapy may be appropriate for young women desiring fertility preservation, while comprehensive laparotomy including total hysterectomy with bilateral salpingo-oophorectomy, omentectomy and lymphonodectomy is recommended for postmenopausal patients (11).

Adjuvant therapy for malignant MCT has not been systematically evaluated due to the rarity of cases. However, early FIGO stage and complete cytoreduction are recognized as key prognostic factors. No standard chemotherapy regimen exists for ovarian apocrine carcinoma; platinum-based therapy has been used with inconsistent outcomes (2–6, 11).

Evidence from breast oncology indicates that AR-positive, triple-negative apocrine carcinomas respond poorly to standard chemotherapy but demonstrate excellent outcomes in early-stage disease, even without adjuvant treatment (7, 9, 13, 14). Trapani et al. (16) further suggested that adjuvant chemotherapy offers no additional benefit in early-stage, AR-positive triple-negative tumors and proposed that antiandrogen therapy could be explored in selected cases (7).

In ovarian apocrine carcinoma, similar principles may apply—complete surgical excision remains the cornerstone of treatment, while adjuvant therapy should be individualized based on stage, receptor profile, and patient preference.

## Conclusion

Ovarian apocrine carcinoma arising in a mature cystic teratoma is an exceptionally rare entity, with few cases reported. This case illustrates that even long-standing, apparently benign teratomas can undergo malignant transformation, particularly in postmenopausal women. Careful clinical follow-up and timely surgical excision remain essential for diagnosis and potential cure. The tumor’s triple-negative but AR- and EGFR-positive profile suggests possible parallels with breast apocrine carcinoma, raising the potential for targeted therapies in advanced or recurrent cases. Long-term monitoring with imaging, serum markers, and future molecular profiling is critical to guide prognosis and personalized treatment.

## Patient perspective

The patient appreciated the clear communication and prompt care, chose to avoid extensive procedures and enjoys the new quality of life after losing over 15 kg.

## Data Availability

The original contributions presented in the study are included in the article/supplementary material, further inquiries can be directed to the corresponding author.
